# Why are olfactory ensheathing cell tumors so rare?

**DOI:** 10.1186/s12935-019-0989-5

**Published:** 2019-10-11

**Authors:** Mariyam Murtaza, Anu Chacko, Ali Delbaz, Ronak Reshamwala, Andrew Rayfield, Brent McMonagle, James A. St John, Jenny A. K. Ekberg

**Affiliations:** 1 Griffith Institute for Drug Discovery, Griffith University, Brisbane, QLD 4111 Australia; 2 Menzies Health Institute Queensland, Griffith University, Southport, QLD 4222 Australia; 3 Clem Jones Centre for Neurobiology and Stem Cell Research, Griffith University, Nathan, 4111 Australia; 4 Department of Otolaryngology-Head and Neck Surgery, Gold Coast University Hospital, 1 Hospital Boulevard, Southport, QLD 4215 Australia

**Keywords:** Glioma, Olfactory nervous system, Schwannoma, Olfactory bulb, Schwann cell, Anterior cranial fossa

## Abstract

The glial cells of the primary olfactory nervous system, olfactory ensheathing cells (OECs), are unusual in that they rarely form tumors. Only 11 cases, all of which were benign, have been reported to date. In fact, the existence of OEC tumors has been debated as the tumors closely resemble schwannomas (Schwann cell tumors), and there is no definite method for distinguishing the two tumor types. OEC transplantation is a promising therapeutic approach for nervous system injuries, and the fact that OECs are not prone to tumorigenesis is therefore vital. However, why OECs are so resistant to neoplastic transformation remains unknown. The primary olfactory nervous system is a highly dynamic region which continuously undergoes regeneration and neurogenesis throughout life. OECs have key roles in this process, providing structural and neurotrophic support as well as phagocytosing the axonal debris resulting from turnover of neurons. The olfactory mucosa and underlying tissue is also frequently exposed to infectious agents, and OECs have key innate immune roles preventing microbes from invading the central nervous system. It is possible that the unique biological functions of OECs, as well as the dynamic nature of the primary olfactory nervous system, relate to the low incidence of OEC tumors. Here, we summarize the known case reports of OEC tumors, discuss the difficulties of correctly diagnosing them, and examine the possible reasons for their rare incidence. Understanding why OECs rarely form tumors may open avenues for new strategies to combat tumorigenesis in other regions of the nervous system.

## Types of glial cells and tumors arising from glial cells

Tumors consisting of glial cells can occur within the central and peripheral nervous system (CNS and PNS, respectively), with those occurring within the CNS referred to as glioma while those within the PNS are referred to as peripheral nerve sheath tumors. Within the CNS, gliomas are the most common intracranial tumors observed in adults and account for 80% of all malignant brain tumors [1, 2]. The main types of CNS glial cells are astrocytes, oligodendrocytes and ependymal cells, whereas the main PNS glial cells include Schwann cells that populate most peripheral nerves, satellite cells in peripheral ganglia and olfactory ensheathing cells (OECs) which are present in the primary olfactory nervous system. The 2016 WHO classification of tumors specifies that tumors are classified according to the genetic profile and histology of the tumor which are more relevant to patient management and treatment options [3]. For example, diffusely infiltrating gliomas are grouped together regardless of whether they are astrocytic or oligodendrocytic. In contrast, within the PNS, peripheral nerve sheath tumors most commonly involve Schwann cells (and are thus termed schwannomas). There are no reports in the literature of tumors from satellite glial cells. In fact, relatively little is known about the function of satellite cells except that they are likely crucial for cell–cell signaling and transmission in sensory ganglia (reviewed in [4]). An extremely rare type of benign peripheral nervous system tumor has been described to arise from OECs [5]. OECs share developmental origin as well as many functional and morphological similarities with Schwann cells (reviewed in [6]), but appear less prone to tumorigenesis than Schwann cells. Indeed, OEC tumors are not mentioned in the WHO classification of tumors of the central nervous system [3] even though OECs are present in the outer layer of the olfactory bulb of the CNS. The existence of OEC tumors is in fact subject to debate as it is very difficult to distinguish OEC tumors from schwannomas.

## Biological functions of OECs in the primary olfactory nervous system

**Fig. 1:**
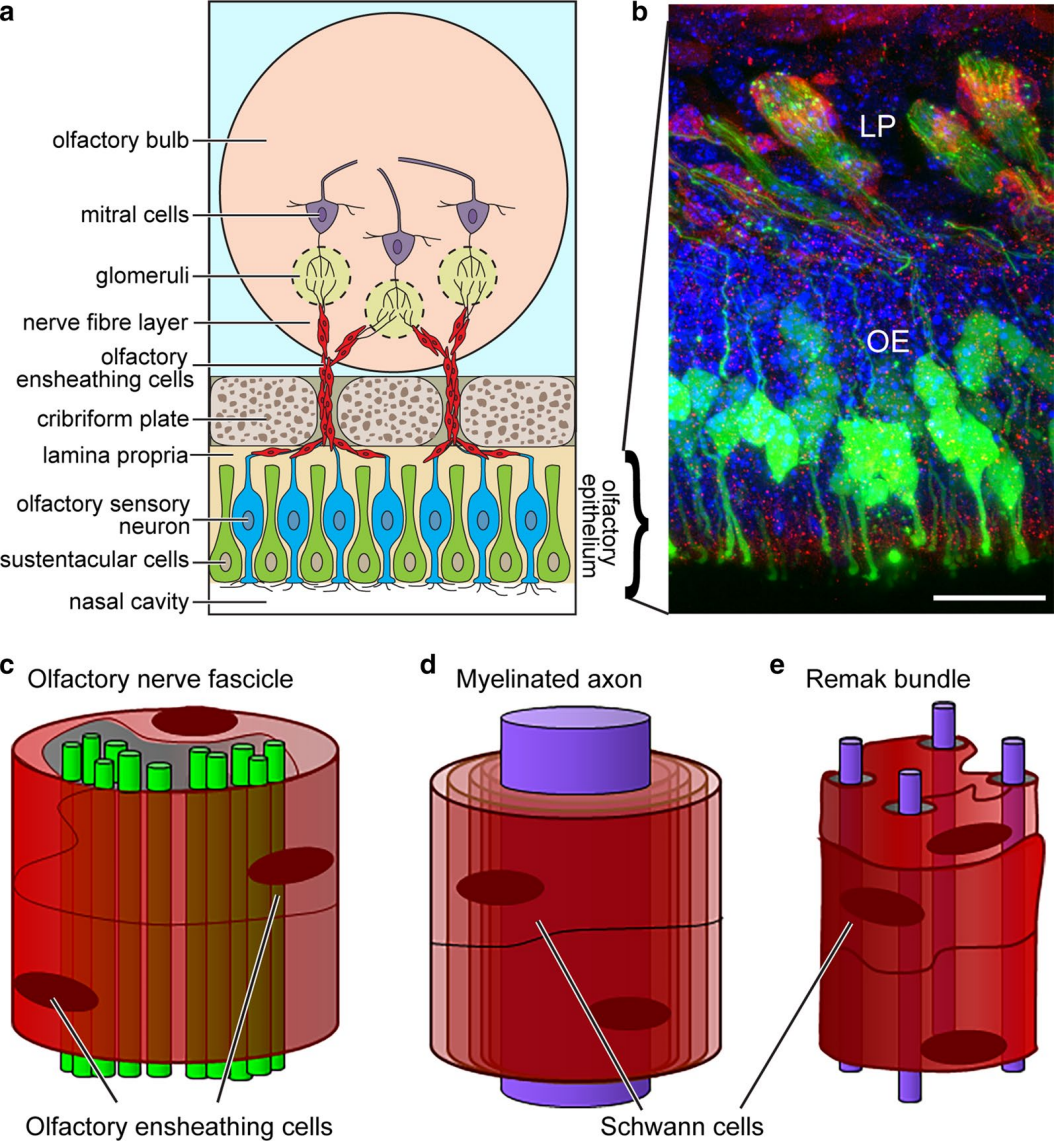
Location of OECs in the primary olfactory nervous system. **a** Schematic of the olfactory nervous system. Olfactory axons extend from cell bodies of olfactory sensory neurons in the olfactory epithelium of the nasal cavity to the olfactory bulb. OECs are present in close contact with these axons in all the way from the periphery to the olfactory bulb in the CNS. In the lamina propria, OECs contribute to axon fasciculation. In the olfactory nerve, fascicles extend towards the bulb, OECs surround axon fascicles. In the outer nerve fibre layer (NFL), OECs contribute to axon defasciculation. In the inner NFL, OECs are involved in axon sorting, refasciculation and targetting to glomeruli based on odorant receptor expression. **b** Shown is an image of a cryostat section from the olfactory epithelium (OE) and lamina propria (LP) of a transgenic mouse (S100βDsRed-OMPZsGreen) in which primary olfactory neurons and OECs express a green and red fluorescent protein, respectively. Primary olfactory neurons (green) within the olfactory epithelium send their axons into the lamina propria where axon fascicles are ensheathed by OECs (red). Scale bar: 35 µm. Blue: nuclei (4′,6-Diamidine-2′-phenylindole dihydrochloride; DAPI). **c** Schematic of an olfactory axon fascicle ensheathed by several OECs surrounding numerous axons. **d**, **e** Arrangement of Schwann cells and axons in peripheral nerves for comparison. **d** A large-diameter axon myelinated by Schwann cells. **e** A Remak bundle in which non-myelinating Schwann cells support small-diameter unmyelinated axons

The primary olfactory nervous system consists of [1] the olfactory nerve extending from the nasal cavity to the olfactory bulb in the brain, and [2] the outermost layer of the olfactory bulb termed the nerve fibre layer (NFL) (Fig. 1a). Olfaction exhibits the strongest association with memory and emotions amongst the senses in humans, and has an important role in distinguishing favourable from non-favourable or potentially dangerous surroundings in other mammals and lower vertebrates. Therefore, olfaction has had massive impact on survival throughout evolution. However, primary olfactory sensory neurons are constantly exposed to irritants, toxins and pathogens entering the nasal cavity. Most likely for this reason, the primary olfactory nervous system has evolved to constantly regenerate itself, and is unique in that it undergoes lifelong neurogenesis. Olfactory sensory neurons live for approximately 1 month in rodents (the exact life-span of human olfactory sensory neurons remains unknown), and 1–3% of neurons are turned over daily [7]. The olfactory sensory neurons are continually replenished from progenitors in the olfactory epithelium. The continuous regeneration of the primary olfactory nervous system is thought to be highly dependent on OECs, which are specialised glial cells with unique neurotrophic properties (reviewed in [6, 8–10]).

Olfactory sensory neurons extend dendrites, on which odorant receptors are localised, to the mucosal surface of the olfactory epithelium, and axons basally into the lamina propria. The axons of olfactory sensory neurons form fascicles (“bundles”), which together constitute the olfactory nerve, extend through the cribriform plate and reach their targets in the olfactory bulb [11–14] (Fig. 1a–c). When the fascicles reach the NFL in the olfactory bulb, the axons defasciculate, sort out and then refasciculate with axons expressing the same odorant receptor [15]. These now uniform fascicles extend to specific targets (glomeruli) in the olfactory bulb; each glomerulus is the target for axons expressing an individual type of odorant receptor [16] (Fig. 1a, b). Thus, throughout life, new axons are continuously finding their way from the cell bodies in the olfactory epithelium all the way to their targets in the olfactory bulb. OECs are present in direct contact with olfactory sensory axons all the way from the lamina propria in the periphery to the NFL of the olfactory bulb. OECs give the olfactory axons structural support and have crucial roles in guiding and regulating the behaviour of the axons, which differ depending on anatomical location [17, 18] (Fig. 1a, b). In the olfactory nerve, OECs ensheath olfactory axon fascicles. The OECs do not myelinate olfactory axons; the fascicles instead consist of many unmyelinated axons surrounded by OECs [18] (Fig. 1c). This contrasts with most peripheral nerves which consist of both myelinated and unmyelinated fibers supported by myelinating and unmyelinating Schwann cells (Fig. 1d, e, respectively); discussed below. In the NFL of the olfactory bulb, OECs are also intimately associated with olfactory axons and are thought crucial for axon defasciculation, sorting and refasciculation [17, 18]. OECs secrete many neurotrophic factors, such as nerve growth factor (NGF), brain-derived neurotrophic factor (BDNF), various neuregulins and other neurotrophins (reviewed in [6, 19–22]). Furthermore, OECs are the primary phagocytes in the olfactory nerve, responsible for clearing axonal debris resulting from the turnover of olfactory neurons and after injury to the olfactory nerve [23–27]. OECs also have important innate immune functions preventing pathogens from invading the CNS via the olfactory nerve [23, 28–31]. Due to their ability to promote growth and survival of neurons, as well as their unique ability to migrate long distances, OECs have been investigated as viable candidates for cell therapies for spinal cord injuries [32–43], neurodegenerative diseases [44–46] and peripheral nerve repair [47–50] with promising but highly variable outcomes.

## Similarities and differences between OECs and Schwann cells

**Fig. 2:**
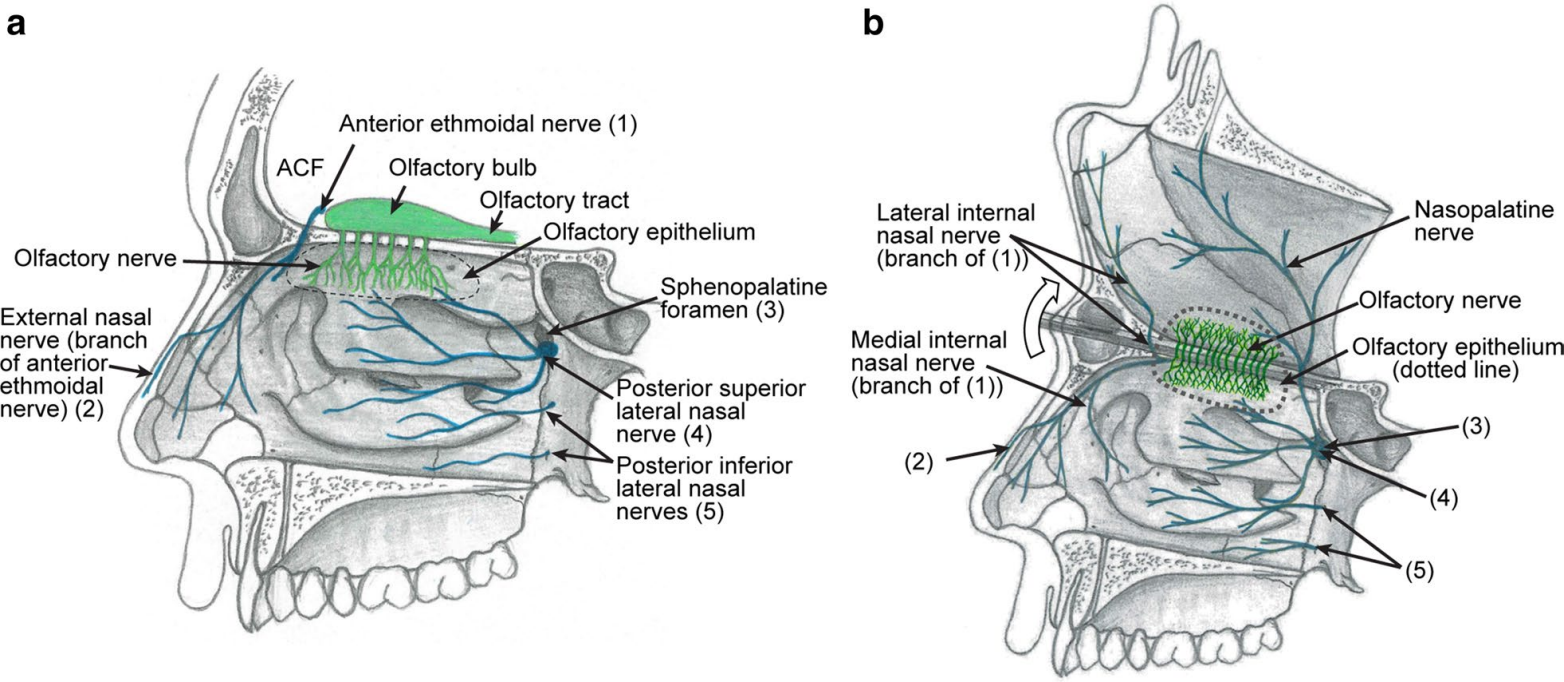
Anatomical location of the trigeminal nerve branches that innervate the nasal cavity. The schematics show sagittal views of **a** the nasal cavity and anterior cranial fossa (ACF), and **b** the nasal cavity with a reflected view of the nasal septum. The olfactory nerve and bulb are shown in green, whereas trigeminal nerve branches are blue. The region of the nasal mucosa in which the cell bodies of olfactory sensory neurons are localised is termed the olfactory epithelium (dotted line). The highly branched olfactory nerve (green) extends from the olfactory epithelium at the roof of the nasal cavity to the olfactory bulb in the anterior cranial fossa. The nasal mucosa is innervated by the nasal branches of the ophthalmic (V1) and maxillary (V2) divisions of the trigeminal nerve (blue). V1 innervates the roof and anterior aspect of the nasal cavity (to the left in both **a** and **b**), and V2 innervates the posterior and lateral aspects of the nasal cavity (to the right in **a**/**b**). The trigeminal fibers are found throughout the epithelium, also interspersed with olfactory nerve fascicles, and convey sensory information (chemosensory, nociceptive, touch, temperature) to the brainstem. The V1 branch innervating the nasal cavity is the anterior ethmoidal nerve ((1) in **a**). The anterior ethmoidal nerve passes closely by the olfactory bulb in the anterior cranial fossa, and gives rise to the external (2) and internal nasal nerves (medial and lateral branches, shown in **b** only). The V2 branches which innervate the nasal cavity are the lateral nasal nerve (posterior superior and posterior inferior branches, (4) and (5), respectively) and the nasopalatine nerve (shown in **b** only). Due to the close proximity of these trigeminal branches with the olfactory nerve/bulb, it is very difficult to distinguish between OEC tumours and schwannomas which arise from, to date, immunologically indistinguishable cells

Schwann cells are the glial cells of most peripheral nerves, including the trigeminal nerve which innervates the nasal cavity; many small trigeminal nerve branches are present in the same anatomical location as the olfactory nerve fascicles (Fig. 2; discussed below). OECs and Schwann cells share developmental origin (the neural crest [51]), as well as many similar morphological and molecular characteristics and functions; both cell types supply structural and neurotrophic support to axons. OECs and Schwann cells have both been considered for cell transplantation therapies, but OECs are considered preferable due to their ability to continuously promote neural regeneration of the olfactory nerve, their superior migratory and phagocytic properties and their ability to interact with astrocytes (reviewed in [6]). The fact that OECs appear more resistant to tumor formation than Schwann cells is another very important reason for why OECs may be more suitable than Schwann cells for transplantation into damaged neural tracts.

In their natural environment, OECs and Schwann cells display strikingly different interactions with axons. Myelinating Schwann cells form insulating membranous myelin sheaths around individual axons with a diameter larger than 1 µm (Fig. 1d). Non-myelinating Schwann cells instead enwrap several small-diameter axons in a bundle termed a Remak bundle (reviewed in [52]) (Fig. 1e). OECs do not myelinate axons but instead ensheathe axon bundles that are typically much larger than a Remak bundle (Fig. 1c) [53]. Furthermore, while OECs continuously phagocytose axonal debris during olfactory nerve regeneration, Schwann cells only do so after injury [54]. Thus, there are crucial differences between OECs and Schwann cells. Differences in glial cell-axon structural arrangement, migratory properties, responses to injury and innate immune functions may be reasons for why OECs appear more resistant to tumor formation than Schwann cells.

## OEC tumors

OEC tumors are found in the anterior cranial fossa, specifically in the olfactory groove, the sagittal sulcus on the inferior surface of each frontal lobe which contains the olfactory bulb and olfactory tract (Fig. 2). These tumors are thought to originate from OECs in the NFL of the olfactory bulb, or from OECs in the olfactory nerve at the merge with the olfactory bulb (see Fig. 1a, b) [5, 55–63]. There is also one report of an OEC tumor originating from the terminal region of the olfactory nerve near the olfactory mucosa [64]. Since their initial identification in 2006 [5], there have been only 10 other reported cases of OEC tumor (Table 1) [5, 55–64]. The average age of patients was 35.6 years, and seven out of the 11 patients were female. The tumors were in general large (the largest reported was 6.5 cm in diameter), and it is likely that the masses can grow to such size before causing severe symptoms due to the large space available in the anterior cranial fossa [60]. Olfactory dysfunction is a common clinical manifestation; seven out of 11 patients reported anosmia or hyposmia (uni- or bilateral), two patients had normal olfaction and two of the studies did not mention olfactory function prior to surgery. Some of the patients had bone erosion of the skull base or ethmoid bone (Table 1). Seizures (5/11 patients) and/or headaches (4/11 patients) were also reported. No signs of neurofibromatosis or cutaneous stigmata were observed in any of the cases. The tumors varied in appearance with some described as greyish-white [56, 58], firm/solid [56, 58, 63], greyish-red/vascular [59, 60], cystic or cystic-solid [5, 55, 57, 59, 61, 64] or with cystic necrosis [60]. Surgical excision was performed as the main treatment. Excision of the tumor appeared curative with short follow-up times and no metastasis reported; outcomes were in general uneventful with 4/11 patients reporting new or recurring anosmia/hyposmia [56, 60, 62, 63]. Typical pathological characteristics of the excised tumors include spindle-shaped cells in fascicles [5, 57, 60–62] adjacent to looser paucicellular areas [61], similar to Antoni A and Antoni B areas, respectively, in schwannoma tumors [65], fibrous cords [56, 57, 60] and distorted nuclei [5, 59, 60, 64]. (Antoni A areas are highly cellular areas with nuclear palisades and associated Verocay bodies; Verocay bodies constitute two stacked rows of elongated palisading nuclei alternating with zones containing cytoplasmic schwannoma cell extensions. Antoni B areas are localised adjacent to Antoni A areas and consist of loosely arranged cells in myxomatous tissue (tissue with mucoid substance) and microcysts [66]). Overall, very little is known about the clinical and immunohistochemical characteristics of OEC tumors, which makes it very difficult to give a definite diagnosis. The immunohisto/cytochemical markers identified in the known cases are summarised in Table 1.

**Table 1:** Features and immunoprofile of the published case reports

Sex/age	Symptoms	Location	Enhancement	Tumour features	Pathology	Outcome	Marker profile	Reference
Female 31	Right-sided anosmia, generalised seizures	Anterior cranial fossa, attached to the olfactory groove	Heterogeneous	Irregular, avascular, cystic-solid, capsulated tumour with calcified nodules. 6.5 cm diameter. Bone erosion	Spindle-shaped cells in a wavy cellular arrangement. Distorted and twisted nuclei	Complete removal. Uneventful	S100 + EMA − Leu7 −	[5]
Male 42	Normal olfactory function, generalised seizures	Anterior cranial fossa (left subfrontal region), arising from the left olfactory bulb	Heterogeneous	Round, cystic-solid fibrous tumour	Spindle-shaped cells, fibrous cords. Curved vesicular nuclei with ill-defined cytoplasmic margins	Complete removal. Uneventful	S100 + EMA – GFAP – SMA – Leu7 –	[57]
Male 32	Olfactory function not mentioned, seizures	Anterior cranial fossa (left frontal base)	Heterogeneous	Round, solid, greyish-white tumour with a glistening appearance and rubbery consistency. 3.6 × 3.3 × 3.9 cm	Spindle-shaped cells, fibrous cords. Ovoid, elongated, normochromatic, comma-shaped nuclei. Interrupted deposits of basal lamina in the cellular membrane	Complete removal. Uneventful.	S100 + (80% of cells) Leu7 – Calretinin – Podoplanin – EMA – GFAP –	[58]
Female 28	Anosmia, focal seizures	Anterior cranial fossa	Heterogeneous	Greyish-white, irregular, cloudy, solid. 4 × 3.5 × 2.5 cm	Well-circumscribed tumour with elongated spindle-shaped cells, fibrous cords. Moderate nuclear pleomorphism	Complete removal. Anosmia	S100 + Synaptophysin + EMA – Leu7 –	[56]
Female 30	Right-sided anosmia, headache	Anterior cranial fossa, intradural, extra-axial space and attached to the right cribriform plate	Homogenous	Round, solid 4 cm diameter.	Cells formed patterns of compact fascicular Antoni A areas (resembling Schwannoma) with palisading nuclei	Complete removal. Olfactory function was not restored	S100 + EMA – Leu7 –	[63]
Female 41	Anosmia, headache	Olfactory mucosa; olfactory cleft extending superiorly to the olfactory groove	Heterogeneous	Irregular, cystic tumour. Bone defect in the skull base	Spindle-shaped cells with eosinophilic cytoplasm and elongated or wavy nuclei with occasional symplastic changes	Subtotal resection. Uneventful	S100 + Neuron-specific enolase + Synaptophysin + (weakly) EMA – Leu7 –	[64]
Male 49	Hyposmia, visual impairment	Anterior cranial fossa	Homogenous	Round, cystic-solid tumour, eroding the right cribriform plate	Unknown	Complete removal. Uneventful	S100 + EMA – Leu7 –	[55]
Male 20	Normal olfaction, headache, generalized seizures	Anterior cranial fossa	Heterogeneous	The tumour grew towards the left olfactory groove and compressed the left frontal cortex. Greyish-red, vascular tumour. Cystic necrosis inside the tumour. 3.4 × 2.6 × 5.0 cm	Spindle-shaped cells were predominantly arranged in compact fascicles or fibrous cords and a few cells were arranged in whorls	Complete removal, hyposmia	Vimentin + S100 + EMA – Leu7 –	[60]
Female 45	Olfactory function not mentioned, foreign body sensation	Anterior cranial fossa.	Heterogeneous	Irregular, cystic tumour 6.2 × 6.0 × 4.0 cm	Spindle-shaped cells. Compact, fascicular Antoni A areas as well as Antoni B areas	Complete removal. Uneventful	S100 + Leu7 – GFAP – EMA –	[61]
Female 34	Hyposmia, dizziness, emotional lability	Anterior cranial fossa	Homogenous	Well-defined cystic, greyish-red mass, 3.1 cm diameter	Spindle cells with eosinophilic protoplasm, tadpole-shaped nucleus	Complete removal. Uneventful	Vimentin + S100 + EMA – GFAP – Leu7 –	[59]
Female 40	Left-sided anosmia, migraine, headaches	Anterior cranial fossa, olfactory groove adjacent to the left inferior anterior frontal lobe	Heterogeneous	3.2 cm diameter	Spindle cell neoplasm characterized by extensive palisading and prominent Antoni A (Verocay bodies) and Antoni B areas	Complete removal. Left-sided anosmia	S100 + Type IV collagen + Leu7 – EMA –	[62]

+: positive; cell is expressing marker; –: negative; cell is not expressing marker; S100: S100 protein (glial marker); Leu7 (CD57 or HNK-1): suggested marker for Schwann cells but not OECs; EMA: Epithelial membrane antigen; GFAP: Glial fibrillary acidic protein; SMA: Smooth muscle actin

## Why is it difficult to distinguish between OEC tumors and schwannomas?

### Overlapping anatomical location of the primary olfactory nervous system and trigeminal nerve branches

Schwannomas (nerve sheath tumors originating from Schwann cells) can arise from any peripheral or cranial nerve in which the glial cells are Schwann cells. The sporadic schwannomas, which are compared to OEC tumors in this review, are distinct to nerve sheath tumors seen in the genetic conditions neurofibromatosis and schwannomatosis which are caused by germline mutations [67]. The most common location for schwannomas is the head and neck; approximately 3–4% of humans exhibit head or neck schwannomas on autopsy [68]. Schwannomas comprise approximately 8% of all intracranial tumors [69]. Malignant schwannomas are uncommon but aggressive and comprise 2% of all sarcomas with a high metastatic potential and poor prognosis [70]. It is very difficult to distinguish OEC tumors from schwannomas and meningiomas, which can be present in the same anatomical areas as OEC tumors (Fig. 2) and cause similar symptoms to OEC tumors, including anosmia [5, 58–60, 64]. In particular, it is difficult to distinguish between OEC tumors and schwannomas, since both tumors arise from glial cells with a shared developmental origin (the neural crest, [51]), as well as many similar morphological and molecular characteristics (reviewed in [6]). The schwannomas that are so easily confused with OEC tumors are usually termed anterior cranial fossa schwannomas or olfactory groove schwannomas (OGS) but can also occasionally be found in the nasal cavity and paranasal sinuses (nasoethmoid schwannomas) [71–75]. These schwannomas are rare; to date, approximately 45 cases (without neurofibromatosis/schwannomatosis) have been reported in the literature [76, 77]. They are thought to originate from Schwann cells of the nasal branches of the ophthalmic (V1) and maxillary (V2) divisions of the trigeminal nerve (Fig. 2) [63, 78, 79]. These branches innervate the olfactory epithelium and underlying lamina propria, and pass closely to the olfactory bulb in the anterior cranial fossa, regions in which OECs are present (Fig. 2).

It has also been suggested that schwannomas can arise from the terminal nerve (cranial nerve zero) [78, 80, 81], a bilateral plexus of unmyelinated fascicles extending from the nasal epithelium via the cribriform plate and medial surface of the olfactory bulbs towards the preoptic hypothalamic area [60]. The terminal nerve branches closely follow and intermingle with olfactory nerve fascicles, and the terminal nerve is often mistaken for the olfactory nerve in post-mortem humans. Whilst this nerve is well documented in many vertebrates, and it has been reported to exist in human embryos since the early 1900s, its existence in the adult human brain was not confirmed until the 1990s [82]. It is thought to have roles in gonadotropin-releasing hormone (GnRH) signaling and the hypothalamic-pituitary–gonadal (HPG) axis, but has also been suggested to be vestigial in adulthood (reviewed in [83]). To date, the cellular nature of the glial cells of this nerve has not been studied, but as the nerve resembles many peripheral nerves, the terminal nerve glial cells are most likely Schwann cells [82, 83]. Further, developmental theories suggest that schwannomas in the nasal cavity/anterior cranial fossa arise from mesenchymal pial cells which transform into Schwann cells, or from aberrant neural crest cells [78, 80, 81, 84, 85]. In summary, OECs and Schwann cells share many similarities and are found in the same anatomical region (Fig. 2); thus, it is very difficult to distinguish between OEC tumors and schwannomas. This has led to speculation on the true origin and identity of schwannomas and OEC tumors.

### Lack of OEC-specific markers

The clinical and radiological features of OEC tumors and trigeminal nerve schwannomas are indistinguishable. Instead, the two types of tumors are usually classified immunocytochemically based on the expression of the marker Leu7 (reviewed in [78]). Leu7, also known as CD57 or HNK-1, is expressed by Schwann cells in sciatic and trigeminal nerves [86, 87], but not by OECs. It has also been reported that cultured human [39, 88] and rat [19] Schwann cells are Leu7-positive whilst OECs are Leu7-negative. Thus, gliomas of the nasal cavity and anterior cranial fossa/olfactory groove that do not express Leu7 are considered to be OEC tumors [5]; in all (11/11) case reports of OEC tumors, the lack of reactivity to Leu7 was used to conclude the diagnosis to be OEC tumor. Lack of Leu7 expression, however, does not necessarily mean that the tumor is definitely an OEC tumor, as ~ 20% of schwannomas are negative for Leu7 [89]. Recently, two cases of schwannoma-like tumors in the anterior cranial fossa were described to be immunonegative for Leu7 but immunopositive for Schwann/2E, a marker for myelinating Schwann cells [90] and some schwannoma tumors [91]. Leu7 is expressed by Schwann cells in early development and then lost; when Schwann cells myelinate axons their expression of Leu7 is again up-regulated (reviewed in [78]). In addition to being expressed by myelinating Schwann cells, Leu7 is present in Schwann cells that have ingested myelin during Wallerian degeneration [86, 92]. However, cultured Schwann cells lose the expression of Leu7 once phagocytosed myelin debris becomes degraded [93]. To date, Leu7 expression has not been detected in human non-myelinating Schwann cells. A study in the adult canine trigeminal nerve showed that whilst myelinating trigeminal Schwann cells express Leu7, non-myelinating Schwann cells do not [93]. One report shows expression of Leu7 in cultured rat non-myelinating Schwann cells [19], but these cells may have been exposed to myelin debris and thus the Leu7 immunoreactivity may have been labelling phagocytosed material. Overall, it is likely that Leu7 expression by non-myelinating Schwann cells is low or non-existent. Thus, tumors arising from non-myelinating Schwann cells may very well be Leu7-negative. The percentage of unmyelinated axons in the various branches of the human trigeminal nerve is not well characterized, except that the majority of axons are myelinated [94]. Counts of unmyelinated fibres are difficult as the axons are closely packed together in groups and it is difficult to distinguish individual axons [95]. Studies from the 1920s show that the trigeminal nerve contains ~ 10% unmyelinated axons in the cat [96], and ~ 20–40% in the dog [95]. The percentage of unmyelinated fibres is estimated to be 12–20% in human motor root [97], but may very well be higher in the sensory root which contains small, nociceptive unmyelinated C-fibers [98]. Regardless, the number of non-myelinating Schwann cells in the trigeminal nerve is significant and it can thus be expected that a significant proportion of schwannomas arise from these cells.

Expression of Leu7 may, conversely, also not necessarily mean that a tumor is a schwannoma and not an OEC tumor. One study shows that some OEC populations in the olfactory bulb of rats are in fact Leu7-positive [19], further rendering Leu7 immunoreactivity as an inappropriate marker to distinguish between the two types of tumors. Therefore, the identification of OEC tumors based solely on the absence of Leu7 immunoreactivity is inconclusive, and diagnostic tests should involve multiple markers rather than reliance on the absence of a single marker. To date, no markers that definitely distinguish between OECs and Schwann cells have been identified (reviewed in [6]). Schwann/2E expression has not yet been characterized in OECs, and regardless, Schwann/2E appears to be, like Leu7, a marker specific for myelinating Schwann cells [90].

Moreover, neoplastic transformation and tumorigenesis is a dynamic process where cells within a tumor may no longer retain the same cellular properties or molecular signature as the cells of origin. In the case of schwannomas, abnormal or lost axon-Schwann cell interactions, including myelination, has been suggested as being implicated in tumorigenesis [99–102] (discussed in more detail below). This, again, highlights the fact that Leu7, or other markers for myelinating Schwann cells, are not appropriate for diagnosis of schwannomas, as loss of expression of these markers is likely to accompany loss of myelination [93]. Due to the difficulties in using Leu7 as a marker of OEC versus Schwann cell tumors, and the lack of a suitable panel of other markers to distinguish between OECs and Schwann cells, the possibility exists that some of the tumors diagnosed as OEC tumors may in fact have originated from Schwann cells. Thus, OEC tumors may be even rarer than the few cases to date reported in the literature.

## Why are OEC tumors so rare?

### Local environment, plasticity and proliferation

Two-way communication between cells and their microenvironment is critical for tissue homeostasis and for tumor growth. According to the seed and soil cancer hypothesis, the fate of tumor-initiating cells (seed) is guided by the presence of favourable microenvironments (soil) [103]. The olfactory nerve is a neurogenic niche where olfactory neurons are replaced throughout life, and where axons continuously extend towards the olfactory bulb [7, 11–14]. The environment is frequently exposed to external insults and there is constant turnover of neurons; thus, this is a uniquely plastic region of the nervous system. Furthermore, OECs effectively respond to widespread injury of the olfactory nerve or olfactory bulb by proliferating [104, 105]. One may assume that a niche so permissive for proliferation is likely to have a higher probability of developing transformed cells, precancerous lesions and tumors. Contrary to this expectation, OEC tumors are extremely rare. It is plausible that the threshold for tumor initiation is higher in this niche (either due to the environment or intrinsic properties of OECs), than in other nervous system regions, so the olfactory nerve can remain permissive for axon growth throughout life. This is to date a speculation, and the cellular and molecular mechanisms involved that may render OECs less susceptible to cancer than other glial cells are unknown. Whilst it is clear that OEC proliferation and differentiation must be tightly regulated, the normal life-span of OECs, and the mechanisms regulating OEC proliferation/differentiation, has not been characterized. In the case of Schwann cell tumors, the local environment appears to influence tumorigenesis, as schwannomas are more common in the vestibular division of the vestibulocochlear nerve (vestibular schwannoma) than in other peripheral nerves [68, 106–108], reasons for which remain unknown. The anatomy of olfactory *versus* trigeminal nerve fascicles may also be of importance. Olfactory nerve fascicles traverse perpendicularly deep “downwards” into the underlying tissue from the olfactory mucosa. In contrast, trigeminal nerve fibers traverse more or less parallel to the nasal mucosal layer (Fig. 2). Therefore, it is possible that Schwann cells exhibit more contact with the superficial lamina propria layer which is exposed to inhaled carcinogens or irritants than OECs. This, in combination with the fact that OECs have evolved to be constantly phagocytic due to the turnover of the olfactory nerve (discussed below) may contribute to the resistance to tumor formation in OECs.

### Cell migration

Cell migration is an essential process during development and throughout life. It is crucial for wound healing, immune surveillance and in pathological processes such as metastasis. The process of cancer metastasis is generally accepted to be due to the detachment and migration of individual cells from a primary tumor that enter the bloodstream or lymphatic vessels and invade distant organs (reviewed in [109, 110]), and in the case of gastrointestinal and ovarian tumors, directly invade the peritoneum [111]. OECs are unique amongst glial cells in that they can migrate along olfactory axons from the PNS into the olfactory bulb (reviewed in [8]). After olfactory nervous system injury, one of the main responses by OECs is to migrate towards the injury site [104, 105]. OECs can also migrate considerable distances into scar tissue after transplantation into the injured spinal cord; this is one of the reasons OECs are such attractive candidates for transplantation therapies [36, 38]. On the cellular level, OEC migration rate is strongly correlated with the number and activity of motile lamellipodia, which are crucial for contact-mediated migration [112–114]. Thus, OECs naturally exhibit strong capacity for migration. To date, the migratory behaviour of neoplastic OECs has not been characterized.

Several factors have been identified to influence OEC migration (reviewed in [8]), in particular glial-derived neurotrophic factor (GDNF), fibulin-3, slit homolog 2 protein (Slit2) and Nogo-66. GDNF is a neurotrophic factor which stimulates OEC lamellipodia and migration [113], and subsequently enhances axon extension [115]. GDNF is positively correlated with malignancy and affects cancer cell metastasis [116, 117]. In contrast, Slit2 and Nogo-66 inhibit migration of OECs [118, 119]. Interestingly, it is reported that Slit2 inhibits neural invasion in cancer [120, 121] and Nogo-66 inhibits the migration of human glioma cells [122]. Fibulin-3 is an extracellular matrix protein and its overexpression inhibits OEC migration and promotes cell proliferation [123]. Fibulin-3 is reported to be upregulated in malignant gliomas and promote glioma growth [124, 125]. While the significance of these factors in the context of OEC tumor formation is unknown, it is possible that the synergism between the different factors and/or the cellular response to the factors may have critical roles in the low incidence of OEC tumors.

### Innate immune functions and inflammation

The olfactory nerve constitutes a direct link between the nasal cavity and the brain, and is therefore a potential route by which microorganisms can enter the CNS. Despite this, microbial CNS invasion via this nerve is rare (reviewed in [126]). We generated transgenic mice in which olfactory neurons and their axons (OMP-ZsGreen mice; [127]) and glial cells (S100β-DsRed mice) [113] express bright fluorescent proteins (Fig. 1b), which allowed us to in detail investigate the cellular arrangement in olfactory nerve fascicles. We also crossed these mice with MacBlue mice [128], in which macrophages, the immune cells of hematopoietic origin that are professional phagocytes, express a fluorescent blue protein. To our surprise, we found that olfactory nerve fascicles were almost completely devoid of macrophages, and we never detected macrophages in direct contact with olfactory axons [27]. Even after olfactory nerve injury [27, 105] or infection with *Burkholderia pseudomallei*, one of the few pathogens capable of infecting the olfactory nerve [129], we found that the number of macrophages in the olfactory nerve was very limited. Instead, we found that OECs are the primary phagocytes responsible for continuously removing cellular debris resulting from olfactory neuron turnover or injury [27, 130]; this has also been shown by others [26, 27]. In addition, OECs also rapidly respond to and phagocytose bacteria, and are now considered essential for the innate immune response against bacterial invasion of the CNS via olfactory nerve fascicles [23, 30]. Thus, OECs are constantly phagocytosing material, mainly debris resulting from regeneration of the olfactory nerve, and also microorganisms. Despite different developmental origins (neural crest *versus* yolk sac myeloid [51, 131, 132]), OECs and microglia appear to share some innate immune functions (constant phagocytic activity, responses to pathogens, cytokine profile [11, 23, 27–30]), which is interesting since microglia rarely form primary tumors. This raises the possibility that the innate immune functions of both OECs and microglia somehow are counteractive to tumor formation.

Interestingly, most of the growth factors and cytokines secreted by CNS glial cells with known implications in tumorigenesis (such as interleukin 6 (IL-6), matrix-metalloproteinase 9 (MMP9), transforming growth factor β (TGF-β), basic fibroblast growth factor (bFGF), epidermal growth factor (EGF) and tenascin-C) are also expressed by OECs [133]. These factors modulate cancer progression via upregulation of tumor cell proliferation, increased migration and immune protection of tumor cells. In this context, it is also surprising that the incidence of OEC tumors is so rare. It is possible that, from an evolutionary perspective, this microenvironment has adapted to preserve olfaction by employing minimal immune response during pathogen clearance. This is clearly reflected in the limited macrophage infiltration of the olfactory nerve. Macrophages are implicated in tumor cell migration, invasion and metastasis making them an integral part of tumorigenesis and tumor-associated inflammation [134]. It is therefore also tempting to speculate that the absence of macrophages from olfactory nerve fascicles could contribute towards the low incidence of OEC tumors. Compartmentalized immune responses within the nasal mucosa of teleost fish have been reported as a strategy to optimize local immune responses without affecting olfactory function [135]. The OEC secretome may have a local protective effect and eliminate the need for infiltration of inflammatory cells, minimizing tissue damage. Efficient clearance of debris, invading microbes and highly regulated immune responses are integral to sustain and preserve olfactory function. However, the cellular and molecular mechanisms that protect the primary olfactory nervous system against infection, whilst limiting inflammation, remain unknown.

Given the phagocytic ability of OECs, it is possible that they also target and phagocytose transformed cells. Microglia and astrocytes can phagocytose glioma cells [136, 137], but this activity is not necessarily very effective (reviewed by [138]). The region comprising the olfactory mucosa, lamina propria and terminal part of the olfactory nerve is continuously exposed to pathogens. The link between pathogens, in particular viruses, and cancer is becoming increasingly evident as pathogens are thought to cause ~ 16% of all cancers [139]. Whilst the specific link between glioma and pathogens remains largely unknown, the possibility exists that OECs may phagocytose not only pathogens but also infected cells (both OECs and other cell types) before the cells can undergo neoplastic transformation as a result of infection. Cells infected with certain viruses, for example, change their expression of “eat me” signals and become phagocytosed by macrophages and neutrophils [140], and it is possible that OECs can also respond to and phagocytose such cells. Oncolytic viruses, which can target and promote apoptosis of glioma cells, also significantly increase microglia-mediated phagocytosis of the tumor cells [141].

### Comparison with Schwann cells and schwannomas

Whilst this review focusses on the differences between OECs and schwannomas not associated with neurofibromatosis/schwannomatosis, it must be mentioned that schwannomas, including sporadic tumors, do have a strong genetic component. It is to date unknown if this is also the case for OEC tumors. In schwannomas associated with neurofibromatosis type 2 (NF2) or schwannomatosis, the *Nf2* gene, which encodes the tumor suppressor protein moesin-ezrin-radixin-like protein (merlin), is mutated [68]. Mutations in the *Nf2* gene also contribute significantly to sporadic schwannomas; 66% of sporadic vestibular schwannomas were found to have mutations in this gene (such mutations are non-germline mutations, occurring in the tumor but not in the germline) [142]. To date, no information is available regarding *Nf2* mutations in spontaneous schwannomas occurring in the same region as OEC tumors. Similarly, the role of merlin in OEC tumors, and in normal OEC biology, remains unknown. In Schwann cells, merlin is involved in a variety of intracellular signaling pathways and cellular functions (proliferation, migration, differentiation and tumorigenesis) [143]. Merlin has crucial roles in peripheral nerve regeneration after injury [144] and mediates interactions between axons and Schwann cells by regulating neuregulin-ErbB signaling [102, 145]. The ErbB receptor tyrosine kinase family is a group of receptor tyrosine kinases (RTKs) consists of four cell surface receptors (ErbB1/EGFR/HER1, ErbB2/HER2, ErbB3/HER3, and ErbB4/HER4). ErbB receptors are mutated or overexpressed in many cancers [146]. In the rat, OECs show distinctly higher expression of ErbB2 and ErbB4, while Schwann cells express primarily ErbB2 and ErbB3 [147–150]. ErbB3, specifically, has been identified as a biomarker for facial schwannomas in Tasmanian devils [151]. In humans, vestibular schwannoma tissues have been shown to exhibit much higher levels of phosphorylated ErbB3 in comparison to healthy paired nerves, and ErB inhibitors have been identified as a novel therapy for malignant schwannomas [152]. Furthermore, ErbB3/HER3 is now emerging as a novel selective therapeutic cancer target [153]. Thus, differences in expression of ErbB receptors, in particular ErbB3, between OECs and Schwann cells may therefore contribute to differential tendency to tumorigenesis between the two types of glial cells. Investigating the roles of merlin in normal OEC biology and in OEC tumor formation, as well as further characterizing the roles of ErbB receptors in peripheral gliomas, is therefore important for understanding potential differences between the two types of tumors.

It is also possible that the reason OEC tumors are rarer than schwannomas are related to differences in the biology of OECs and Schwann cells, or to the microenvironment in which the cells exist. The fact that the olfactory nerve continuously regenerates, whilst peripheral nerves only regenerate after injury, may also be crucial; peripheral nerve injuries, which lead to accumulation of myelin debris, have been implicated in tumorigenesis [99–102]. As discussed earlier, the olfactory nerve is a highly plastic environment in which OECs constantly phagocytose debris and respond to microorganisms. In contrast, peripheral nerves populated by Schwann cells do not undergo regeneration unless they have been injured, and Schwann cells in their natural environment do not often encounter microorganisms. After peripheral nerve injury, Schwann cells lose their contact with axons, become phagocytic “repair” Schwann cells [54] and proliferate [154]. Disrupted axon-Schwann cell contact has been implicated in schwannomagenesis [99–102]. Axonal injury has been shown to contribute towards a persistent regenerative “repair Schwann cell” response promoting schwannomagenesis, in particular in combination with mutations in the *Nf2* gene [101]. As OECs continuously play an active role in neural regeneration, it is possible that they are less prone to pathological injury-related responses than Schwann cells. Again, this may be due to intrinsic cellular properties, or to the local environment of the olfactory nerve. We have demonstrated that the phagocytic activity of OECs but not Schwann cells can be strongly stimulated with curcumin [155, 156]. This suggests that the phagocytic machinery in the two cell types is regulated by different mechanisms, and perhaps OECs exhibit much greater scope for up-regulation of phagocytic activity in response to nerve injury or infections than Schwann cells.

Denervated Schwann cells produce chemotactic cues that attract macrophages [157], which infiltrate peripheral nerve injury sites and have an essential role in Wallerian degeneration and regeneration [158]. As macrophages are strongly involved in tumorigenesis (reviewed in [159]), it is possible that macrophages also have a role in schwannoma formation. One study shows a strong correlation between schwannomagenesis and the presence of macrophages, in particular M2-polarised macrophages [101]. Interestingly, aspirin intake, which limits inflammation and macrophage infiltration, has been correlated with slowed growth of schwannomas [160]. As discussed earlier, macrophages are mostly absent from the olfactory nerve fascicles, and macrophage invasion is very limited even after widespread injury. It is possible that differences in inflammatory responses between the olfactory nerve and other peripheral nerves populated by Schwann cells are crucial determinants of the likelihood of tumor formation. Furthermore, significant differences between OECs and Schwann cells in responses to bacteria have been identified: OECs, but not Schwann cells, respond to gram-negative bacteria or lipopolysaccharide (LPS) with nuclear translocation of NFκβ and secretion of the chemokine Gro [30], suggesting that OECs exhibit more pronounced innate immune functions than Schwann cells. Interestingly, schwannoma cells are characterized by abnormal activation of NFκβ, which is normally suppressed by merlin, resulting in secretion of pro-inflammatory cytokines and macrophage recruitment (reviewed in [161]). Thus, it is possible that unique regulatory mechanisms in OECs, but not Schwann cells, allow the cells to respond to pathogens, clear cell debris and secrete pro-inflammatory cytokines without causing excessive inflammation, macrophage infiltration and increased risk of tumor formation.

## Conclusions

OEC tumors are difficult to distinguish from schwannomas, as the two types of tumors are found in the same anatomical location, cannot be distinguished radiologically (CT/MRI) and originate from cells with numerous similarities. Currently, OEC tumors and schwannomas are classified based on Leu7 expression [5, 55–64]; however, this marker is not suitable for distinguishing between the two glial cell tumor types. It is therefore essential to further characterize molecular differences between OECs/OEC tumors and Schwann cells/schwannomas. Regardless, OEC tumors are rare. The reasons for this are currently unknown but may relate to the fact that the primary olfactory nervous system constantly undergoes regeneration. OECs have evolved to support this regeneration by becoming a dynamic and responsive population of cells which perform distinct physiological functions in a context-dependent manner. OECs have unique functions in maintaining homeostasis in the olfactory system and they rapidly adapt and respond to new environmental cues. OECs are active phagocytes and innate immune cells, constantly removing cellular debris and protecting the olfactory nerve against microbial invasion. Schwann cells, on the other hand, are not continuously phagocytosing debris or responding to microorganisms. Injury to peripheral nerves populated by Schwann cells leads to demyelination and macrophage attraction, processes suggested to contribute to schwannoma. In contrast, the olfactory nerve is not myelinated, macrophages are largely absent from nerve fascicles and macrophage invasion after injury or infection is highly limited. These differences between peripheral nerves and the primary olfactory nervous system may be related to the likelihood of tumor formation. It is also possible that the local environment near the olfactory epithelium, nerve and bulb is not very permissive to tumor formation, which would also explain why schwannomas in this region are rarer than, for example, in the vestibular nerve. Regardless, the fact that OECs appear resistant to neoplastic transformation is a further indication for using these cells in transplantation therapies for nervous system injuries.

In summary, the reasons for why OEC tumors are so rare remain unknown. Possible reasons include intrinsic cellular and molecular properties in OECs that (1) prevent transformation into tumor cells or limit responses to oncogenic stimuli, (2) tightly regulate proliferation and migration, and (3) allow phagocytosis of debris and microorganisms whilst limiting inflammatory responses. The local dynamic environment and structure of the olfactory nerve (in particular, lack of myelin) may also contribute. Future studies investigating interactions between OECs and immune cells, in particular macrophages, will shed more light on the role of OECs in inflammation and cancer. Understanding the functions of OECs under normal physiological conditions, as well as how they behave in inflammatory and tumor environments, can offer insights into mechanisms initiating gliomagenesis. If there are unique factors that render OECs more resistant to tumor formation than other glial cells, these can be exploited in the future to provide therapeutic benefits to non-OEC microenvironments in the fight against cancer.

## Data Availability

Not applicable.

## Abbreviations

OECs: olfactory ensheathing cells
CNS: central nervous system
PNS: peripheral nervous system
NFL: nerve fibre layer
NGF: nerve growth factor
BDNF: brain derived neurotrophic factor
OGS: olfactory groove schwannomas
GnRH: gonadotropin releasing hormone
HPG: hypothalamic pituitary gonadal
GDNF: glial derived neurotrophic factor
Slit2: slit homolog protein 2
IL6: interleukin 6
MMP9: matrix metalloproteinase 9
TGF-β: transforming growth factor β
bFGF: basic fibroblast growth factor
EGF: epidermal growth factor
NF-2: neurofibromatosis type 2
LPS: lipopolysaccharide
